# Amelioration of Motor Performance and Nigrostriatal Dopamine Cell Volume Using a Novel Far-Infrared Ceramic Blanket in an A53T Alpha-Synuclein Transgenic Parkinson’s Disease Mouse Model

**DOI:** 10.3390/cimb45120613

**Published:** 2023-12-06

**Authors:** Frederick Robert Carrick, Luis Sebastian Alexis Valerio Hernandez, Kiminobu Sugaya

**Affiliations:** 1 College of Medicine, University of Central Florida, Orlando, FL 32827, USA; ksugaya@ucf.edu; 2 Burnett School of Biomedical Science, University of Central Florida, Orlando, FL 32827, USA; valeriohlsa@hms.harvard.edu; 3 MGH Institute for Health Professions, Boston, MA 02129, USA; 4 Centre for Mental Health Research in Association, University of Cambridge, Cambridge CB2 1TN, UK; 5 Department of Neurology, Carrick Institute, Cape Canaveral, FL 32920, USA

**Keywords:** A53T alpha-synuclein transgenic mice, Parkinson’s disease, substantia nigra, striatum, far-field infrared ceramic, rotarod

## Abstract

We had attended a Parkinson’s Disease (PD) patient for a non-healing wound who reported a marked decrease in his hand tremor and freezing of gait when his wound was exposed to a ceramic far-field infrared (cFIR) blanket. PD is the most frequent motor disorder and the second most frequent neurodegenerative disease after Alzheimer’s Disease (AD). The tremor, rigidity, and slowness of movement associated with Parkinson’s disease (PD) affect up to 10 million people throughout the world, and the major contributing factor to the pathogenesis of PD is the accumulation and propagation of pathological α-synuclein (α-Syn) and the death of dopaminergic cells in the Nigrostriatal system. Efforts to slow or stop its spreading have resulted in the development and use of dopaminergic drug replacement therapy. Unfortunately, there is a loss of about 70–80% of substantia nigral dopaminergic neurons in patients by the time they are diagnosed with PD, and various dopaminergic drugs provide only temporary relief of their motor symptoms. There are limitations in treating PD with many conventional medications, necessitating a combination of pharmaceutical and non-pharmacological therapy as an essential adjunct to better address the health and welfare of PD patients. We used male adult A53T alpha-synuclein transgenic mice exposed to a ceramic far-infrared blanket. Motor activity was assessed using the rotarod apparatus, and mouse brains were examined to quantify the fluorescence intensities of the immunostained samples. A53T alpha-synuclein transgenic mice had a significantly shorter time stay on the rotating bar than the wild-type mice (B6C3H). The rotarod performance was significantly improved in A53T alpha-synuclein transgenic mice exposed to cFIR as well as B6C3H healthy wild mice exposed to cFIR. There was a significant statistical and substantive increase in the cellular composition of the Striatum and substantia nigra of cFIR-treated mice. Improvement in motor performance is seen in PD mice and wild mice and is associated with increases in cell volume in the substantia nigra and striatum after treatment.

## 1. Introduction

We had attended a Parkinson’s disease (PD) patient for a non-healing wound that reported a marked decrease in his hand tremor and freezing of gait when his wound was exposed to a ceramic far-field infrared (cFIR) blanket. Wound healing was accelerated in a mouse model exposed to that cFIR ceramic blanket [1], and we were intrigued about our patient’s amelioration of PD motor function and wanted to test this technology’s effectiveness using a PD mouse model.

The tremor, rigidity, and slowness of movement associated with PD affect up to 10 million people worldwide [2,3]. PD was first described as a shaking palsy by Dr. James Parkinson in 1817, and since that time, it has been well recognized as a progressive disease that affects not only patients but their families and caregivers as well [4,5]. Non-motor symptoms, including psychosis, depression, sleep disturbance, anxiety, fatigue, cognitive decline, pain, and the motor symptoms of bradykinesia, rigidity, instability, lack of coordination, and tremor [3] characterize PD. The treatment outcomes of PD are disappointing, leaving people with this second most common neurodegenerative disorder in desperate situations [6]. 

The significant contributing factor to the pathogenesis of PD is the accumulation and propagation of pathological α-synuclein (α-Syn) and the formation of toxic α-Syn oligomers with α-Syn immunohistochemistry, considered the gold standard in the neuropathological evaluation of PD [7]. α-Syn is encoded by the SNCA gene localized on the long arm of chromosome 4 (Chr 4q22.1), with Lewy body inclusions in the SN associated with nigrostriatal degeneration characterizing classical PD [8]. A systematic spread of α-Syn is seen as Lewy pathology progressing throughout the nervous system in stages of localization, correlating with no clinical expression of PD in the early stages and clinical expression realized in the later stages [9]. The seminal work by Braak and colleagues demonstrated Lewy pathology progression to serial induction sites beginning in the dorsal motor nucleus of the glossopharyngeal and vagal nerves and anterior olfactory nucleus. From there, the progression of α-Syn affects the less vulnerable nuclear grays and the brain stem, with cortical involvement beginning with the anteromedial temporal mesocortex. The high-order sensory association and prefrontal areas follow, leading to further α-Syn first-order sensory association/premotor areas and primary sensory/motor fields [9].

The striatum receives extensive afferent input from the midbrain, thalamus, and cortex, such that its neural synchrony and firing rate are changed [10,11] in PD. Historically, striatal dopamine (DA) depletion has been central to our understanding of the motor symptoms of PD, even though DA acts throughout the basal ganglia, not just in the striatum [12]. In the Albin model, PD was hypothesized to increase basal ganglia output from changes in striatal projection neuron subpopulations [13]. The Albin model hypothesized that the projection systems in the striatum were composed of a direct pathway that promoted movement, and an indirect pathway suppressing movement (Albin et al., 1989). Skilled motor behaviors require bihemispheric coordination and participation of striatal outputs originating from two neuronal groups identified by distinctive expression of D1 or D2 dopamine receptors [14]. Degeneration of the substantia nigra and its tract to the striatum occurs in PD patients, and is associated with motor severity on the Unified Parkinson’s Disease Rating Scale (UPDRS) [15,16]. Skilled movements occur when output pathways work together, but toxic conversion and accumulation of alpha-synuclein protofibrils arise in PD [17].

Efforts to slow or stop dopaminergic cell death have resulted in the development and use of dopaminergic drug replacement therapy. Unfortunately, there is a loss of about 70–80% of substantia nigral dopaminergic neurons in patients by the time they are diagnosed with PD, and various dopaminergic drugs provide only temporary relief of their motor symptoms [18,19]. The hyperdopaminergic symptoms of dyskinesia, the dystonic or hyperkinetic involuntary movements associated with dopamine replacement, complicate the treatment of PD [20,21]. The loss of mesencephalic dopaminergic neurons innervating the striatum has been considered one of the leading causes of PD principal motor symptom pathology, with levodopa-induced dyskinesia occurring through pharmacological overstimulation of striatal DA receptors [22]. Levodopa-responsive parkinsonism and progressive loss of axonal function may be triggered by nigral dopaminergic neuronal mitochondrial complex 1 (MCI) [23] disorders. Human-like parkinsonism can be caused by MCI dysfunction in mice, producing motor dysfunction after the loss of the nigral dopamine [24]. 

Progressive neurodegeneration in PD occurs because of synaptic dysfunction and synaptopathy, with deregulated dopamine caused by accumulating α-Syn altering the size of synaptic vesicle pools [25]. Nigrostriatal dopamine pathway malfunction is largely responsible for the clinical pathology of PD because of the loss of dopaminergic neurons in the substantia nigra (SN), resulting in aberrant function of medium spiny neurons, their target cells in the dorsal striatum [4]. The organelle dysfunction associated with α-Syn accumulation and toxicity results in mitochondrial dysfunction to endolysosomal defects as well as impaired functionality of the Golgi and endoplasmic reticular stress [26]. Dopaminergic neuronal loss occurs due to mitochondrial dysfunction causing inflammation in PD that is associated with a loss of autophagy and clearing of damaged mitochondria and other damaged structures [27]. Mitochondrial dysfunction occurs early in the midbrain of A53T mice, mediated by the impairment of NCX3 protein expression in astrocytes and neurons that trigger neuroinflammation in the striatum, contributing to the progression of PD [28]. Impaired mitochondrial function is central to PD pathogenesis, yet these defects are reversible, encouraging treatments that might repair mitochondria [29]. The wavelength emitted by the cFIR used in wound healing is associated with therapeutic benefits, including the stimulation of healing, prevention of tissue necrosis, increases in mitochondrial function, improvement in blood flow, and tissue oxygenation, while also acting as an anti-inflammatory agent in a wide range of medical applications [30]

## 2. Materials and Methods

### 2.1. Ethical Approval

All animal care and experimental procedures were performed following the guidelines and ethical standards set forth by the National Institutes of Health Guide for the Care and Use of Laboratory Animals, and under a protocol approved by the Institutional Animal Care and Use Committee (IACUC) at the University of Central Florida (PROTO202000030).

### 2.2. Biosafety, Biosecurity, and Institutional Safety Procedures

This study was conducted under the biosafety and biosecurity guidelines of the University of Central Florida.

### 2.3. Animals

A total of 60 male adult B6C3H and 60 heterozygous A53T alpha-synuclein transgenic mice (B6; C3-Tg(Prnp-SNCA*A53T)83Vle/J) were used in this investigation. These mice were obtained by breeding a pair of A53T alpha-synuclein transgenic mice purchased from Jaxon Laboratory (Bar Harbor, ME) in our laboratory. PD motor phenotypes appear at 8 months of age in mice expressing A53T human alpha-synuclein [31], and our mice were nine months old, with bradykinesia at the start of the experiment. They were housed under standard laboratory conditions with a 12 h light/dark cycle, constant temperature of 22 ± 2 °C, and humidity of 50 ± 10%. They had access to food and water ad libitum. 

### 2.4. Far-Field Infrared Ceramic Blanket

Their home cages (containing four mice per cage) were placed on a cFIR blanket to expose the mice to ceramic-induced far-infrared radiation. Some 30 B6C3H and 30 heterozygous A53T alpha-synuclein transgenic mice allocated to the cFIR groups were placed in cages with cFIR blankets and lived there for four weeks, which was the extent of the investigation. Some 30 B6C3H and 30 heterozygous A53T alpha-synuclein transgenic mice were allocated to the control groups and were placed in cages without blankets and lived there for four weeks. We used the cIFR wavelength patented and manufactured by Gladiator Therapeutics, LLC. (Whitehall, Pennsylvania). The blankets were composed of ceramic samples made into a shape of one-third circumference cutout of a 12 mm long cylindrical tube, with 15 mm ID (inner diameter) and 30 mm OD (outer diameter). The base mixture of the ceramic FIR-emitting oxides contains, by weight, 20% silicate, 20% alumina, 24% zirconia, and other minority oxides such as sodium monoxide, potassium oxide, ferric oxide, chromic oxide, nickel oxide, and cobalt oxide. The mixture of metal oxides, bonding agents, catalysts, and stabilizers was press-molded to the desired shapes and sintered in a furnace above 1100 °C. All ceramic samples were arranged in an array formation secured with a silicone rubber (polydimethylsiloxane) mold compound.

### 2.5. Rotarod

This study assessed motor activity using the rotarod apparatus (Orchid Scientific, Acceleration Model, Maharashtra, India). The device consists of a rotating rod with a diameter of 3 cm and a length of 30 cm, divided into separate lanes by plastic dividers. Mice were randomly assigned to the Wild and A53T control groups, and active cFIR Wild and A53T treatment groups (n = 10 per group) for the rotarod test to compare their motor performance. Before the test, animals were acclimated to the testing room for at least 30 min, and trained on the rotarod for three consecutive days to familiarize them with the task. During the test session, each mouse was placed on the rod facing away from the experimenter. The rotarod was started at an initial speed of 5 RPM, with a gradual increase of 0.1 RPM/s. The latency to fall was recorded for each animal, up to 180 s. Each animal underwent three trials, with a resting period of at least 15 min between trials.

### 2.6. Immunohistochemistry

After the four-week study, all animals were anesthetized using a combination of ketamine (100 mg/kg) and xylazine (10 mg/kg). The anesthesia adequacy was assessed through the toe pinch and blink reflex. The animals were euthanized at the end of the cardiac ventricle perfusion with a solution of 10% sucrose and 4% paraformaldehyde. Following perfusion, the mouse brains were removed and placed in buffered 4% paraformaldehyde fixative (pH 7.4) containing 20% sucrose overnight. Any tissue not immediately processed was flash-frozen in liquid nitrogen and stored at −80 °C. All brains were sliced into coronal sections of 20 µm thickness using a cryostat-microtome, with serial sections collected in 0.1 M phosphate-buffered saline (PBS) for use. Any unused sections were stored free-floating at −80 °C in a cryoprotectant solution containing 15% glycerol in 0.1 M phosphate buffer until they were processed for immunohistochemistry. The brain sections of 3 mice per group were transferred to a solution of 0.1 M PBS containing 0.25% Triton X-100 (PBST) for 30 min, followed by blocking in PBST containing 3% donkey normal serum for 1 h. Next, the sections were incubated with a tyrosine hydroxylase antibody (185, ThermoFisher Scientific, Waltham, MA, USA, MA1-24654, 1:40) overnight at 4 °C. After rinsing with PBS, a donkey anti-mouse antibody conjugated to rhodamine IgG (Jackson IR Laboratories, Inc., West Grove, PA, USA) was added at a 1:300 dilution in PBST for a 2 h incubation at room temperature in the dark. The brain sections were thoroughly washed with PBS before mounting on a slide and coverslipping with Vectashield containing DAPI (Vector Laboratories, Inc., Burlingame, CA, USA). One representative section per brain was utilized in the quantification of the immunofluorescent imaging.

### 2.7. Quantification of Immunofluorescent Imaging

We used the software Image J (NIH) to quantify the fluorescence intensities of the immunostained samples. The images were converted into an 8-bit digital scale, and a threshold of regions of interest (ROI) for all the cells from the images was set manually to obtain the mean gray value corresponding to the fluorescence intensities. We selected ROIs with a minimum of 1 nuclei and a minimum of 50 ROIs. Then, we normalized the averaged data using the fluorescence intensities from DAPI signaling, and expressed the averaged data as a ratio of fluorescence intensities from the average measured ROI relative to DAPI.

Finally, the corresponding graphics were generated using the software PRISM.

### 2.8. Statistical Analysis

Statistical analysis was performed with STATA 18, StataCorp, College Station, Texas and PRISM 7, GraphPad Software, Boston, MA. We used multiple one-way ANOVA tests with Bonferroni’s multiple comparisons as a post hoc procedure to correct the family-wise error rate, following an ANOVA to find evidence of statistical significance with an alpha < 0.05 and a power of 80%. We used Cohen’s d for effect sizes to establish any substantive significance. 

## 3. Results

### 3.1. Rotarod Motor Performance

Shapiro–Wilk tests did not show evidence of non-normality. We calculated the mean latency to fall (in seconds) from the rotarod for each mouse, and compared the mean latencies between the control and experimental groups, as shown in Table 1.

**Figure 1:**
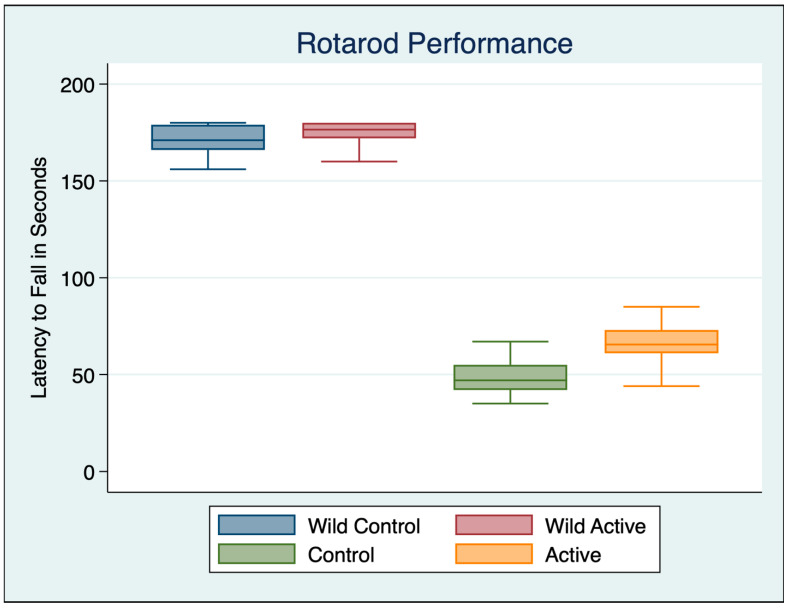
Rotarod performance A53T alpha-synuclein transgenic mice and wild C57BL/6 mice.

A one-way ANOVA was performed with a Bonferroni multiple comparisons test adjustment to compare the effect of the four different groups on the rotarod performance test, as shown in Table 2, Figure 1.

### 3.2. Substantia Nigra

Shapiro–Wilk tests did not show evidence of non-normality. We compared the immunofluorescent intensity of the substantia nigra for each mouse, and compared the mean immunofluorescent intensity between the control and experimental groups, as shown in Table 3.

**Figure 2:**
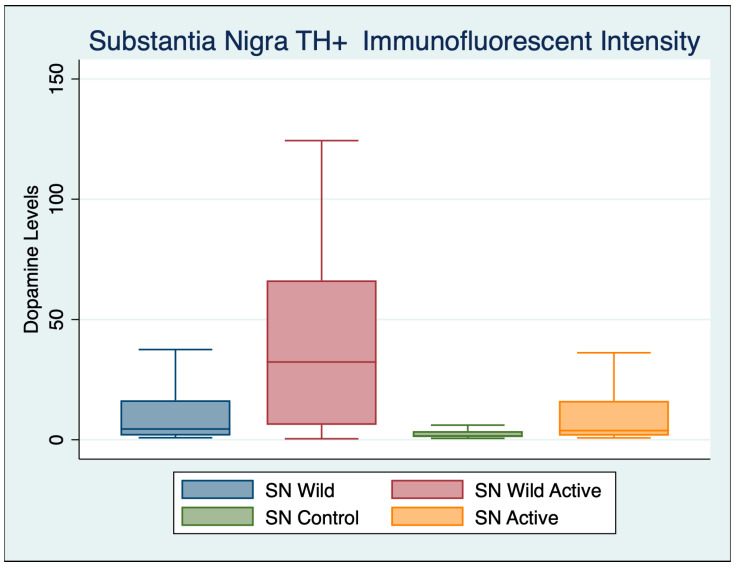
Substantia nigra TH+ immunofluorescent intensity for dopamine levels demonstrates high statistical and substantively significant differences between both wild and A53T control and active mice.

**Figure 3:**
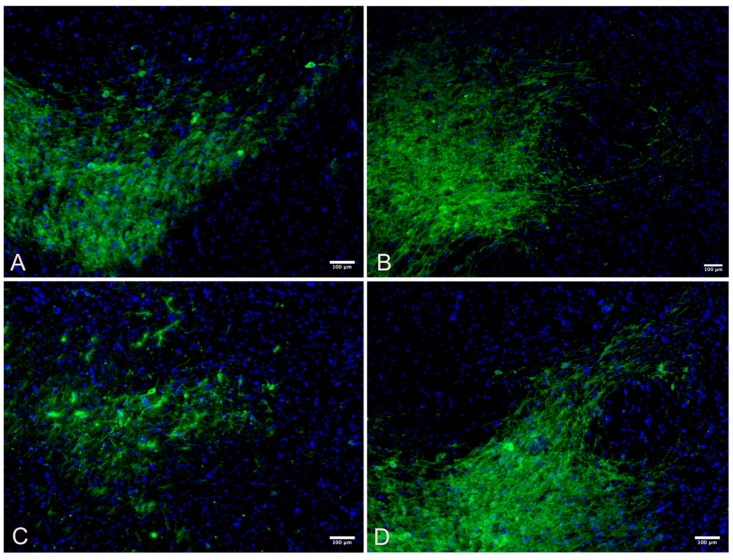
Substantia nigra TH+ immunofluorescent intensity, stained with tyrosine hydroxylase (green) for dopamines and nuclei of the cells counterstained with DAPI (blue). (**A**) Wild Control; (**B**) Wild Active; (**C**) Control; (**D**) Active.

A one-way ANOVA was performed with a Bonferroni multiple comparisons test adjustment to compare the substantia nigra immunohistochemistry of the four different groups as shown in Table 4, Figure 2 and Figure 3.

### 3.3. Striatum

Shapiro–Wilk tests did not show evidence of non-normality. We compared the immunofluorescent intensity of the striatum for each mouse and the mean immunofluorescent intensity between the control and experimental groups, as shown in Table 5.

**Figure 4:**
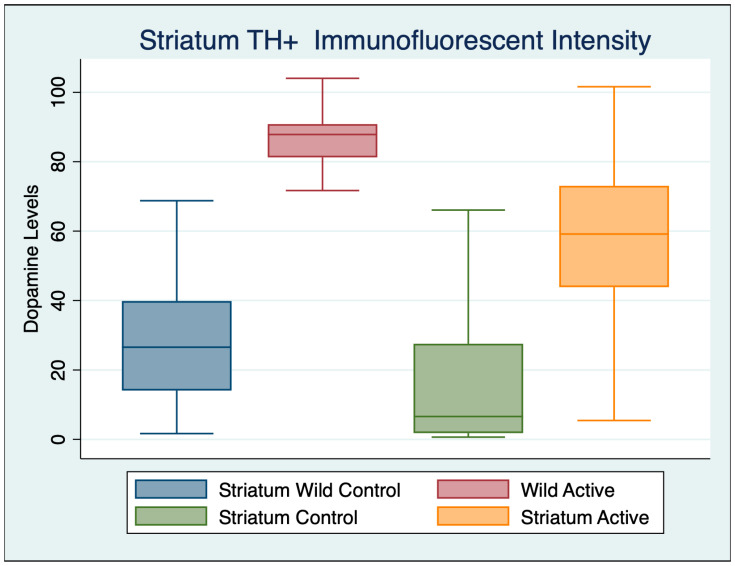
Striatum TH+ immunofluorescent intensity for dopamine levels demonstrates high statistical and substantively significant differences between both wild and A53T control and active mice.

**Figure 5:**
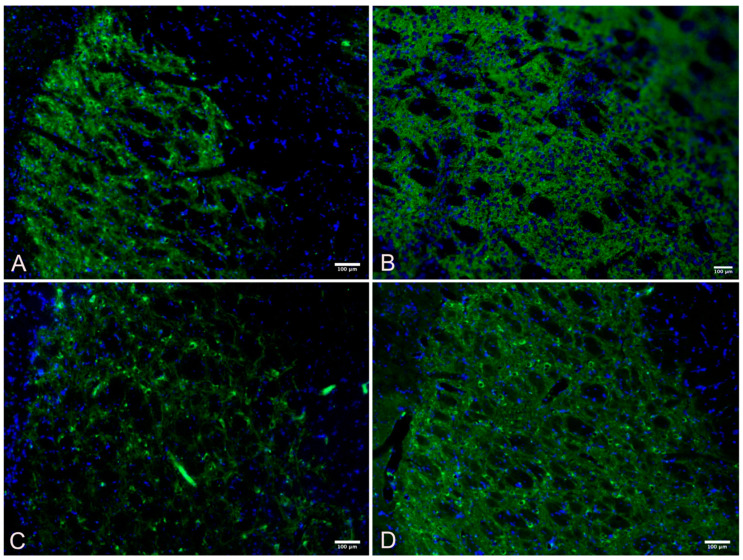
Striatum TH+ immunofluorescent intensity, stained with tyrosine hydroxylase (green) for dopamine, and nuclei of the cells counterstained with DAPI (blue). (**A**) Wild Control; (**B**) Wild Active; (**C**) Control; (**D**) Active.

A one-way ANOVA was performed with a Bonferroni multiple comparisons test adjustment to compare the striatum immunohistochemistry of the four different groups, as shown in Table 6, Figure 4 and Figure 5.

## 4. Discussion

The rotarod data confirmed the bradykinesia of PD by comparing the rotarod performance of the healthy control wild mice to the A53T control mice. Wild mice could stay on the rotarod significantly longer than the A53T control mice. There was a 71.16% longer latency to fall time observed in wild controls compared to A53T controls, but the time difference fell to a 63.52% greater time in the wild controls compared to the A53T active group. The A53T cFIR-treated (active) mice showed improved motor performance, decreasing bradykinesia and increasing their time on the rotarod by 25.88% compared to the A53T control mice. Interestingly, there was also a 2.37% improvement in motor function in the healthy wild active mice treated with cFIR, representing a moderate substantively significant effect size, although not statistically significant. 

This study has demonstrated that cFIR results in significantly improved motor performance in PD mice, with associated increases in the volume of cells in both the substantia nigra and striatum. It also increases the motor performance of wild mice without PD. Exercise in PD mouse models is well established as a modifier of brain and behavior function [32]. Further, treadmill training might slow down the progression of Parkinson’s disease by attenuating mitochondrial respiratory deficiency and neural mitochondrial quality-control dysregulation [33]. Yet, we observed highly significant changes in the brain and in behavior in an A53T mouse model without any exercise. These findings are exciting because of mobility and balance pathology limits in elderly PD patients who might find benefits and increased motor performance with cFIR treatment. In contrast to many potential therapies for PD, the cFIR blanket has significant and novel merit, with none of the toxicity or immune rejection concerns that accompany pharmaceuticals and other biomaterials. 

One of the hallmarks of PD is a loss of dopaminergic neurons in the substantia nigra. Dopamine depletion is central to our understanding of PD, and the development of a variety of dopaminergic drugs has offered only temporary relief of motor symptoms, but with the unfortunate complications of dyskinesia [12,18,19,20,21]. Levodopa is still the gold standard for the treatment of PD, yet levodopa therapy-induced dyskinesia and OFF symptoms remain unresolved [34]. 

We found a significant difference in the dopaminergic cell volume in the substantia nigra between healthy wild control mice and A53T control mice, as demonstrated by TH+ immunofluorescent intensity. There were 62.37% more cells in the wild controls compared to the A53T controls. After cFIR treatment, the A535T active mice had a 69.80% significant increase compared to the A53T controls, and had 19.75% more cells than the healthy wild control mice. Incredibly, the wild active mice treated with cFIR also had increased cell counts, and were found to have 72.83% more cells than the wild control mice.

The capacity for neurogenesis in the adult mammalian brain is extremely limited and highly restricted to a few regions, which greatly impedes functional restoration and neuronal regeneration after neuronal loss in PD [35]. There are limitations in treating PD with many conventional drugs, necessitating a combination of pharmaceutical and non-pharmacological therapy as an essential adjunct to better address the health and welfare of PD patients [3]. The use of polychromatic light in PD treatment increased attention to circadian rhythm approaches as an adjuvant to dopamine replacement therapies in 1996, and is still being considered today [36]. The concept of using both light and FIR stimulation is not new in the treatment of PD, although most studies have suffered from methodological problems. Many neurotherapeutic interventions have been developed for the treatment of PD based upon molecular features, although there is a need for the integration of the basics into clinical science applications, such as those demonstrated in this investigation [37].

We found 62.37% more cells in the striatum of wild controls compared to A53T controls, as demonstrated by TH+ immunofluorescent intensity. However, after cFIR treatment. we found 51.45% more cells in A53T active than wild control mice, and a 69.80% increase in the cells of A53T active mice compared to A53T control mice. Incredibly, we found a 72.83% increase in cells of wild active mice treated with cFIR, compared with wild control mice. All of the multiple group comparisons were highly statistically significant with associated high substantively significant effect sizes, except for comparisons between wild controls and A53T controls that demonstrated a moderate effect size, as did the wild active compared to control A53T mice.

The cFIR blanket is simple to use anywhere, as it is autonomous and without a power supply [38,39,40,41,42]. Recent studies suggest that FIR induces nitric oxide [43], a key molecule with an established role in central nervous system function, such that its dysregulation is associated with PD [44]. Oxidative stress further contributes to the pathology of Parkinson’s disease, while nitric oxide, associated with eustress, might ease the harmful effect [45]. There are also proinflammatory factors in PD that may be mitigated by the anti-inflammatory effects of nitric oxide induced by cFIR [46]. Complicating its function, the nigrostriatal dopaminergic pathway progressively degenerates in PD, with a characteristic loss of neurons in the substantia nigra pars compacta. This phenomenon is reversed by the use of a cFIR ceramic in our investigation [47]. FIR also induces heat shock proteins (HSPs) [48] that participate in the degradation of misfolded and aggregated proteins, identified as a pivotal role in the pathogenesis of PD [49]. HSPs prevent protein misfolding while inhibiting the apoptosis involved in PD pathogenesis [50]. Small HSPs can mutate and cause neurodegenerative disorders, whereas they can also protect against neurodegeneration caused by protein aggregation [51]. 

Mitochondrial dysfunction is a known causal factor in the pathogenesis of neurodegenerative disorders including Parkinson’s disease, Huntington’s disease, Alzheimer’s disease, amyotrophic lateral sclerosis, Friedreich’s ataxia and Charcot–Marie–Tooth disease [52]. Mitochondrial dysfunction occurs in Parkinson’s disease, as high-basal-energy-demanding dopaminergic neurons are susceptible to energy fluctuations that result in disorders of cellular bioenergetics that can be improved by near-infrared (NIR) photobiomodulation enhancement of mitochondrial function [53]. The cFIR improvements in cell volume and performance are probably due in part to changes in cellular bioenergetics. The core pathogenesis of PD is mitochondrial dysfunction, and amelioration of its function is a crucial target for therapy [54]. The outcomes reported in this study suggest that cFIR treatments result in mitochondrial biogenesis with a probable reduction in reactive oxygen species and protection of the respiratory chain. Glucose metabolism and mitochondrial function are improved by far-infrared rays (FIR) due to their vibration and radiation frequencies, with a potential for positive clinical outcomes in the treatment of metabolic diseases [55]. Mitochondrial functional improvement can result in cellular volume changes and plasticity in the striato-nigral system, which we have observed. Further, bio-active materials such as in the cFIR blanket also probably improve mitochondrial oxidative respiration in skeletal muscle and invigorate mitophagy [56], which may contribute to the increased motor performance demonstrated in this investigation. The effects of the cFIR on motor performance appear not to be limited to skeletal muscle because of the robust changes in cell volume in the striatum and substantia nigra that we observed. 

Wu and colleagues [57] identify essential and complex molecular machinery associated with polarized stem cell migration, remodeling the cytoskeleton as in our observations, which is beyond the scope of this report. The remodeling of the substantia nigra and striatum was significant in our investigation. This suggests that cFIR may be involved in a polarized microtubule array, established by microtubule facilitation of cell migration sustaining coordinated cell movements [58,59]. 

Rhythmic oscillation patterns facilitate the cellular microtubular generation of electric and mechanical patterns that impact biomolecular recognition, which boosts our inherent ability for self-healing [60]. The use of cFIR may affect oscillatory patterns, resulting in the accelerated MSC-promoted changes in cellular volumes of the substantia nigra and striatum we observed. Further investigations will be needed to confirm this mechanism. However, the enhancement of the migration of MSCs may have resulted in the cytoarchitectonic changes we observed in this investigation. Using cFIR in cellular biology and medical applications may be promising in treating PD and developing animal and human models. It is also exciting to observe that motor performance in non-PD wild mice is also improved with exposure to cFIR therapy, leading to many potential applications in sports training and the activity of daily living. This technology needs no direct or battery power source and is entirely autonomous and noninvasive, making its application possible in any environment.

## 5. Limitations

The use of A53T alpha-synuclein transgenic mice may not fully represent the complexity of PD in human subjects. It will be important to design trials using the cFIR in human PD subjects in order to answer questions about the translational relevance of the findings of this study. Rotarod testing was utilized as the sole measure of motor activity in this study, and may not capture the full spectrum of motor deficits associated with PD. Future studies should include more testing of motor performance, utilizing additional standardized testing. Additional molecular and cellular assays may complement our immunohistochemistry analysis and provide a more comprehensive understanding of the effects of cFIR treatment on the neuropathology of PD. We used only male mice in this study, and therefore cannot comment on any sex-related differences. We did not identify any stages in Parkinson’s disease advancement where this protocol may be most useful. 

## 6. Conclusions

Motor performance in a PD mouse model is improved by exposure to a novel cFIR device that is autonomous without needing a power supply. An improvement in motor performance was observed in A53T mice and healthy wild mice, and is associated with increased cell volume in the substantia nigra and striatum after treatment. The significant increase in cell volume in healthy wild mice may represent a unique method for the prevention of neurodegeneration in Parkinson’s disease, while the increase in cell volume in the nigrostriatal dopaminergic pathway in A53T mice may represent a viable treatment option in Parkinson’s patients. Further applications and rigorous clinical trials using this technology will further contribute to the potential utilization of a novel therapy in the treatment of PD and in improving human motor performance. This is of the utmost importance considering the limited outcomes and iatrogenesis associated with most currently available therapies. We recommend investigation of cFIR therapy in other neurodegenerative diseases. 

## Figures and Tables

**Table 1:** Rotarod descriptive statistics.

Variable	Obs	Mean	Std. Dev.	Min	Max
Wild Control	30	169.800	10.480	137	180
Wild Active	30	173.933	6.817	160	180
Control	30	48.967	9.891	35	78
Active	30	66.067	9.336	44	85

**Table 2:** ANOVA rotarod performance with Bonferroni’s multiple comparisons test.

ANOVA Multiple Comparisons F (3, 116) = 1551 *p* < 0.001	Mean Diff	95% CI	*p*	Cohen’s d
Wild Control vs. Wild Active	−4.133	−10.54 to 2.269	0.5145	0.4676
Wild Control vs. Control	120.8	114.4 to 127.2	<0.001	11.8588
Wild Control vs. Active	103.7	97.33 to 110.1	<0.001	10.4523
Wild Active vs. Control	125	118.6 to 131.4	<0.001	14.7119
Wild Active vs. Active	107.9	101.5 to 114.3	<0.001	13.1955
Control vs. Active	−17.1	−23.5 to −10.7	<0.001	1.7780

**Table 3:** Descriptive statistics substantia nigra TH+ immunofluorescent intensity.

Variable	Obs	Mean	Std. Dev.	Min	Max
Wild Control	102	10.903	14.428	0.848	94.010
Wild Active	65	40.136	36.569	0.410	124.367
Control	103	4.103	7.176	0.572	56.719
Active	97	13.587	22.989	0.778	106.737

**Table 4:** Substantia nigra immunohistochemistry with Bonferroni’s multiple comparisons test.

ANOVA Multiple Comparisons F (3, 446) = 50.07 *p* < 0.001	Mean Diff	95% CI	*p*	Cohen’s d
Wild Control vs. Wild Active	−29.67	−37.71 to −21.63	<0.001	1.1500
Wild Control vs. Control	6.764	0.2375 to 13.29	0.038	0.5976
Wild Control vs. Active	−3.811	−10.5 to 2.874	0.790	0.1406
Wild Active vs. Control	36.43	28.41 to 44.46	<0.001	0.3545
Wild Active vs. Active	25.86	17.71 to 34.01	<0.001	0.9095
Control vs. Active	−10.57	−17.24 to −3.914	<0.001	0.3428

**Table 5:** Descriptive statistics striatum TH+ immunofluorescent intensity.

Variable	Obs	Mean	Std. Dev.	Min	Max
Wild Control	92	27.672	17.706	1.673	85.779
Wild Active	62	86.381	7.731	51.204	104.041
Control	77	19.955	25.800	0.650	101.263
Active	48	56.996	22.921	5.439	101.619

**Table 6:** Striatum immunohistochemistry with Bonferroni’s multiple comparisons test.

ANOVA Multiple Comparisons F (3, 271) = 203.1	Mean Diff	95% CI	*p*	Cohen’s d
Wild Control vs. Wild Active	−59.83	−68.21 to −51.45	<0.001	4.035203
Wild Control vs. Control	14.43	6.192 to 22.68	<0.001	0.354508
Wild Control vs. Active	−22.39	−30.37 to −14.41	<0.001	1.493189
Wild Active vs. Control	74.26	65.71 to 82.81	<0.001	0.354508
Wild Active vs. Active	37.44	29.14 to 45.74	<0.001	1.81406
Control vs. Active	−36.82	−44.98 to −28.66	<0.001	1.497221

## Data Availability

All data generated or analyzed during this study are included in this published article.

## References

[1] Carrick F.R., Valerio L.S.A., Gonzalez-Vega M.N., Engel D., Sugaya K. (2021). Accelerated Wound Healing Using a Novel Far-Infrared Ceramic Blanket. Life.

[2] Armstrong M.J., Okun M.S. (2020). Diagnosis and Treatment of Parkinson Disease: A Review. JAMA.

[3] Sivanandy P., Leey T.C., Xiang T.C., Ling T.C., Wey Han S.A., Semilan S.L.A., Hong P.K. (2021). Systematic Review on Parkinson’s Disease Medications, Emphasizing on Three Recently Approved Drugs to Control Parkinson’s Symptoms. Int. J. Environ. Res. Public Health.

[4] DeMaagd G., Philip A. (2015). Parkinson’s Disease and Its Management: Part 1: Disease Entity, Risk Factors, Pathophysiology, Clinical Presentation, and Diagnosis. Pharm. Ther..

[5] Parkinson J. (2002). An essay on the shaking palsy. 1817. J. Neuropsychiatry Clin. Neurosci..

[6] Kujawska M., Jodynis-Liebert J. (2018). Polyphenols in Parkinson’s Disease: A Systematic Review of In Vivo Studies. Nutrients.

[7] Li Y., Wang T., Meng L., Jin L., Liu C., Liang Y., Ren L., Liu Y., Liu Y., Liu S. (2023). Novel naturally occurring autoantibodies attenuate α-synuclein pathology in a mouse model of Parkinson’s disease. Neuropathol. Appl. Neurobiol..

[8] Lee V.M., Trojanowski J.Q. (2006). Mechanisms of Parkinson’s disease linked to pathological alpha-synuclein: New targets for drug discovery. Neuron.

[9] Braak H., Del Tredici K., Rüb U., de Vos R.A., Jansen Steur E.N., Braak E. (2003). Staging of brain pathology related to sporadic Parkinson’s disease. Neurobiol. Aging.

[10] Chen H., Lei H., Xu Q. (2018). Neuronal activity pattern defects in the striatum in awake mouse model of Parkinson’s disease. Behav. Brain Res..

[11] Choi K., Holly E.N., Davatolhagh M.F., Beier K.T., Fuccillo M.V. (2019). Integrated anatomical and physiological mapping of striatal afferent projections. Eur. J. Neurosci..

[12] Surmeier D.J., Zhai S., Cui Q., Simmons D.V. (2023). Rethinking the network determinants of motor disability in Parkinson’s disease. Front. Synaptic Neurosci..

[13] Albin R.L., Young A.B., Penney J.B. (1989). The functional anatomy of basal ganglia disorders. Trends Neurosci..

[14] Lopez-Huerta V.G., Denton J.A., Nakano Y., Jaidar O., Garcia-Munoz M., Arbuthnott G.W. (2021). Striatal bilateral control of skilled forelimb movement. Cell Rep..

[15] Lin C.P., Knoop L.E.J., Frigerio I., Bol J., Rozemuller A.J.M., Berendse H.W., Pouwels P.J.W., van de Berg W.D.J., Jonkman L.E. (2023). Nigral Pathology Contributes to Microstructural Integrity of Striatal and Frontal Tracts in Parkinson’s Disease. Mov. Disord..

[16] Zhang Y., Burock M.A. (2020). Diffusion Tensor Imaging in Parkinson’s Disease and Parkinsonian Syndrome: A Systematic Review. Front. Neurol..

[17] Hashimoto M., Rockenstein E., Masliah E. (2003). Transgenic models of alpha-synuclein pathology: Past, present, and future. Ann. N. Y. Acad. Sci..

[18] Ásgrímsdóttir E.S., Arenas E. (2020). Midbrain Dopaminergic Neuron Development at the Single Cell Level: In vivo and in Stem Cells. Front. Cell Dev. Biol..

[19] Dauer W., Przedborski S. (2003). Parkinson’s disease: Mechanisms and models. Neuron.

[20] Peng Q., Zhong S., Tan Y., Zeng W., Wang J., Cheng C., Yang X., Wu Y., Cao X., Xu Y. (2019). The Rodent Models of Dyskinesia and Their Behavioral Assessment. Front. Neurol..

[21] Geibl F.F., Henrich M.T., Oertel W.H. (2019). Mesencephalic and extramesencephalic dopaminergic systems in Parkinson’s disease. J. Neural Transm..

[22] Zhai S., Shen W., Graves S.M., Surmeier D.J. (2019). Dopaminergic modulation of striatal function and Parkinson’s disease. J. Neural Transm..

[23] Cornejo-Olivas M., Wu L., Noyce A. (2022). Disruption of Mitochondrial Complex I Induces Progressive Parkinsonism. Mov. Disord..

[24] González-Rodríguez P., Zampese E., Stout K.A., Guzman J.N., Ilijic E., Yang B., Tkatch T., Stavarache M.A., Wokosin D.L., Gao L. (2021). Disruption of mitochondrial complex I induces progressive parkinsonism. Nature.

[25] Bridi J.C., Hirth F. (2018). Mechanisms of α-Synuclein Induced Synaptopathy in Parkinson’s Disease. Front. Neurosci..

[26] Vrijsen S., Vrancx C., Del Vecchio M., Swinnen J.V., Agostinis P., Winderickx J., Vangheluwe P., Annaert W. (2022). Inter-organellar Communication in Parkinson’s and Alzheimer’s Disease: Looking Beyond Endoplasmic Reticulum-Mitochondria Contact Sites. Front. Neurosci..

[27] Mani S., Sevanan M., Krishnamoorthy A., Sekar S. (2021). A systematic review of molecular approaches that link mitochondrial dysfunction and neuroinflammation in Parkinson’s disease. Neurol. Sci..

[28] Di Martino R., Sisalli M.J., Sirabella R., Della Notte S., Borzacchiello D., Feliciello A., Annunziato L., Scorziello A. (2021). Ncx3-Induced Mitochondrial Dysfunction in Midbrain Leads to Neuroinflammation in Striatum of A53t-α-Synuclein Transgenic Old Mice. Int. J. Mol. Sci..

[29] Milanese C., Payán-Gómez C., Galvani M., Molano González N., Tresini M., Nait Abdellah S., van Roon-Mom W.M.C., Figini S., Marinus J., van Hilten J.J. (2019). Peripheral mitochondrial function correlates with clinical severity in idiopathic Parkinson’s disease. Mov. Disord..

[30] Walski T., Dabrowska K., Drohomirecka A., Jedruchniewicz N., Trochanowska-Pauk N., Witkiewicz W., Komorowska M. (2019). The effect of red-to-near-infrared (R/NIR) irradiation on inflammatory processes. Int. J. Radiat. Biol..

[31] Giasson B.I., Duda J.E., Quinn S.M., Zhang B., Trojanowski J.Q., Lee V.M. (2002). Neuronal alpha-synucleinopathy with severe movement disorder in mice expressing A53T human alpha-synuclein. Neuron.

[32] da Costa Daniele T.M., de Bruin P.F.C., de Matos R.S., de Bruin G.S., Maia Chaves C.J., de Bruin V.M.S. (2020). Exercise effects on brain and behavior in healthy mice, Alzheimer’s disease and Parkinson’s disease model-A systematic review and meta-analysis. Behav. Brain Res..

[33] Nhu N.T., Cheng Y.J., Lee S.D. (2021). Effects of Treadmill Exercise on Neural Mitochondrial Functions in Parkinson’s Disease: A Systematic Review of Animal Studies. Biomedicines.

[34] Prasad E.M., Hung S.Y. (2021). Current Therapies in Clinical Trials of Parkinson’s Disease: A 2021 Update. Pharmaceuticals.

[35] Wang F., Cheng L., Zhang X. (2021). Reprogramming Glial Cells into Functional Neurons for Neuro-regeneration: Challenges and Promise. Neurosci. Bull..

[36] Willis G.L., Armstrong S.M. (2023). Fine-tuning the circadian system with light treatment for Parkinson’s disease: An in-depth, critical review. Rev. Neurosci..

[37] Choonara Y.E., Pillay V., Du Toit L.C., Modi G., Naidoo D., Ndesendo V.M.K., Sibambo S.R. (2009). Trends in the molecular pathogenesis and clinical therapeutics of common neurodegenerative disorders. Int. J. Mol. Sci..

[38] Huang Z., Tian J., Yu B., Xu Y., Feng Q. (2009). A bone-like nano-hydroxyapatite/collagen loaded injectable scaffold. Biomed. Mater..

[39] Janarthanan G., Kim I.G., Chung E.J., Noh I. (2019). Comparative studies on thin polycaprolactone-tricalcium phosphate composite scaffolds and its interaction with mesenchymal stem cells. Biomater. Res..

[40] Kim H.Y., Yu Y., Oh S.Y., Wang K.K., Kim Y.R., Jung S.C., Kim H.S., Jo I. (2019). Far-Infrared Irradiation Inhibits Adipogenic Differentiation and Stimulates Osteogenic Differentiation of Human Tonsil-Derived Mesenchymal Stem Cells: Role of Protein Phosphatase 2B. Cell. Physiol. Biochem..

[41] Wang X., Ma B., Xue J., Wu J., Chang J., Wu C. (2019). Defective Black Nano-Titania Thermogels for Cutaneous Tumor-Induced Therapy and Healing. Nano Lett..

[42] Zhang Z., Dai Q., Zhang Y., Zhuang H., Wang E., Xu Q., Ma L., Wu C., Huan Z., Guo F. (2020). Design of a Multifunctional Biomaterial Inspired by Ancient Chinese Medicine for Hair Regeneration in Burned Skin. ACS Appl. Mater. Interfaces.

[43] Kim S., Park H.T., Soh S.H., Oh M.W., Shim S., Yoo H.S. (2019). Evaluation of the immunobiological effects of a regenerative far-infrared heating system in pigs. J. Vet. Sci..

[44] Iova O.M., Marin G.E., Lazar I., Stanescu I., Dogaru G., Nicula C.A., Bulboacă A.E. (2023). Nitric Oxide/Nitric Oxide Synthase System in the Pathogenesis of Neurodegenerative Disorders-An Overview. Antioxidants.

[45] Jomova K., Raptova R., Alomar S.Y., Alwasel S.H., Nepovimova E., Kuca K., Valko M. (2023). Reactive oxygen species, toxicity, oxidative stress, and antioxidants: Chronic diseases and aging. Arch. Toxicol..

[46] Huang Z., Hu B., Xiang B., Fang H., Zhang B., Wang Y., Zhuo Y., Deng D., Wang X. (2023). Biomimetic Biomembrane Encapsulation and Targeted Delivery of a Nitric Oxide Release Platform for Therapy of Parkinson’s Disease. ACS Biomater. Sci. Eng..

[47] Wang L., Dan Q., Xu B., Chen Y., Zheng T. (2023). Research progress on gas signal molecular therapy for Parkinson’s disease. Open Life Sci..

[48] Kimura I., Yamamoto T., Nakamura K., Uenishi T., Asai T., Kita M., Kanamura N. (2018). Effects of far infrared radiation by isotropic high-density carbon on the human oral mucosa. Arch. Oral Biol..

[49] Zhao Z., Li Z., Du F., Wang Y., Wu Y., Lim K.L., Li L., Yang N., Yu C., Zhang C. (2023). Linking Heat Shock Protein 70 and Parkin in Parkinson’s Disease. Mol. Neurobiol..

[50] Aridon P., Geraci F., Turturici G., D’Amelio M., Savettieri G., Sconzo G. (2011). Protective role of heat shock proteins in Parkinson’s disease. Neurodegener. Dis..

[51] Vendredy L., Adriaenssens E., Timmerman V. (2020). Small heat shock proteins in neurodegenerative diseases. Cell Stress Chaperones.

[52] Chaturvedi R.K., Flint Beal M. (2013). Mitochondrial diseases of the brain. Free Radic. Biol. Med..

[53] Foo A.S.C., Soong T.W., Yeo T.T., Lim K.L. (2020). Mitochondrial Dysfunction and Parkinson’s Disease-Near-Infrared Photobiomodulation as a Potential Therapeutic Strategy. Front. Aging Neurosci..

[54] Zheng Q., Liu H., Zhang H., Han Y., Yuan J., Wang T., Gao Y., Li Z. (2023). Ameliorating Mitochondrial Dysfunction of Neurons by Biomimetic Targeting Nanoparticles Mediated Mitochondrial Biogenesis to Boost the Therapy of Parkinson’s Disease. Adv. Sci..

[55] Seo Y., Kim Y.W., Lee D., Kim D., Kim K., Kim T., Baek C., Lee Y., Lee J., Lee H. (2021). Far-infrared rays enhance mitochondrial biogenesis and GLUT3 expression under low glucose conditions in rat skeletal muscle cells. Korean J. Physiol. Pharmacol..

[56] Lee D., Kim Y.W., Kim J.H., Yang M., Bae H., Lim I., Bang H., Go K.C., Yang G.W., Rho Y.H. (2015). Improvement Characteristics of Bio-active Materials Coated Fabric on Rat Muscular Mitochondria. Korean J. Physiol. Pharmacol..

[57] Wu X., Shen Q.-T., Oristian D.S., Lu C.P., Zheng Q., Wang H.-W., Fuchs E. (2011). Skin stem cells orchestrate directional migration by regulating microtubule-ACF7 connections through GSK3β. Cell.

[58] Watanabe T., Noritake J., Kaibuchi K. (2005). Regulation of microtubules in cell migration. Trends Cell Biol..

[59] Kodama A., Karakesisoglou I., Wong E., Vaezi A., Fuchs E. (2003). ACF7: An essential integrator of microtubule dynamics. Cell.

[60] Facchin F., Canaider S., Tassinari R., Zannini C., Bianconi E., Taglioli V., Olivi E., Cavallini C., Tausel M., Ventura C. (2019). Physical energies to the rescue of damaged tissues. World J. Stem Cells.

